# Desmoplastic fibroma in a child: a 9-year follow-up case report

**DOI:** 10.1186/s12891-024-07454-6

**Published:** 2024-04-20

**Authors:** Yaokai Lu, Wei Lan, Qiangchu Wu, Yi Fu, Shengyuan Lan, Xixiong Wang, Xuwei Huang, Lu Ye

**Affiliations:** Department of Orthopaedic Traumatology, Qinzhou First People’s Hospital, No. 5 Mingyang Street, Qinzhou City, P. R. China

**Keywords:** Desmoplastic fibroma, Tumor resection, Free vascularized fibular proximal epiphyseal transfer, Arthrodesis

## Abstract

**Background:**

Desmoplastic fibroma is an extremely rare primary bone tumor. Its characteristic features include bone destruction accompanied by the formation of soft tissue masses. This condition predominantly affects individuals under the age of 30. Since its histology is similar to desmoid-type fibromatosis, an accurate diagnosis before operation is difficult. Desmoplastic fibroma is resistant to chemotherapy, and the efficacy of radiotherapy is uncertain. Surgical excision is preferred for treatment, but it entails high recurrence. Further, skeletal reconstruction post-surgery is challenging, especially in pediatric cases.

**Case presentation:**

Nine years ago, a 14-year-old male patient presented with a 4-year history of progressive pain in his left wrist. Initially diagnosed as fibrous dysplasia by needle biopsy, the patient underwent tumor resection followed by free vascularized fibular proximal epiphyseal transfer for wrist reconstruction. However, a histological examination confirmed a diagnosis of desmoplastic fibroma. The patient achieved bone union and experienced a recurrence in the ipsilateral ulna 5 years later, accompanied by a wrist deformity. He underwent a second tumor resection and wrist arthrodesis in a single stage. The most recent annual follow-up was in September 2023; the patient had no recurrence and was satisfied with the surgery.

**Conclusions:**

Desmoplastic fibroma is difficult to diagnose and treat, and reconstruction surgery after tumor resection is challenging. Close follow-up by experienced surgeons may be beneficial for prognosis.

## Background

Desmoplastic fibroma (DF) was initially described by Jaffe in 1958 [1] as intraosseous desmoids characterized by the formation of abundant collagen fibers by tumor cells. In 2020, the World Health Organization classified DF as a fibrogenic tumor with local invasiveness composed of bland spindle cells set in abundant collagen [2]. The typical microscopic constituents are proliferative fibroblasts and collagen fibers with different proportions in different areas [3]. DF is rare, accounting for 0.11% of all primary bone tumors [4], with an incidence of approximately 2.5 cases in every 100 million population [5]. A total of 271 cases were reported until 2013 [6], and only a few case reports have been published in recent years. DF often occurs in the first three decades of life with an unclear etiology and no statistical difference in the incidence between male and female patients. DF usually affects the metaphyses of long, flat, and craniofacial bones and is rare in small bones [7, 8].

DF can cause pain, dysfunction, and even pathological fractures at the lesion site. Surgery is considered the primary management approach; however, the recurrence rate is up to 48% [9]. Since surgical excision can lead to bone defects, reconstruction surgery can be challenging, particularly in cases involving large bone or joint defects. If the tumor invades the growth plate, restoration of longitudinal growth is also considered in addition to skeletal reconstruction, as it means a more complex surgery and more complications. Although the cases that occur in children are extremely rare, 9 years ago, we performed a wide excision for a child with a DF located in his left distal radius. To the best of our knowledge, this is the first case of a DF in a child that originated from the radius and had a recurrence in the ipsilateral ulna. We believe the present case can provide valuable insights and lessons, especially regarding surgical method selection and management of complications.

## Case presentation

**Fig. 1:**
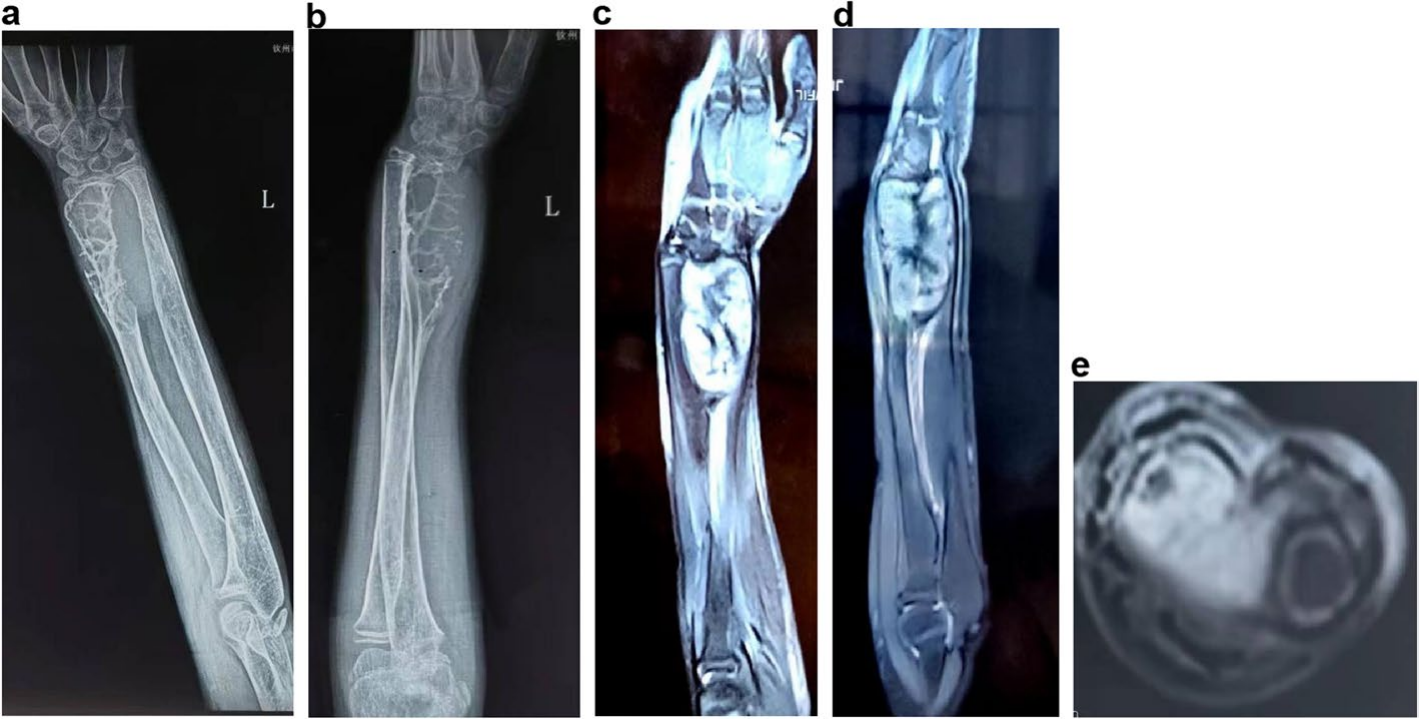
**a** Anteroposterior (AP) and **b** lateral radiographs reveal extensive osteolytic lesions in the distal radius; internal pseudo-trabeculations are observed. Magnetic resonance imaging (MRI) shows a large mass with a clear and smooth rim invading the radial metaphysis and carpal articular surface. **c** Coronal view. **d** Lateral view. **e** AP views

In December 2013, a 14-year-old boy presented to our hospital with a 4-year history of progressive pain in his left wrist; he had no history of preceding trauma. On examination, we observed localized swelling, tenderness, and limitations in the wrist movement. Neurological and vascular examination findings were normal. Anteroposterior and lateral radiographs of the left forearm revealed extensive osteolytic lesions in the distal radius accompanied by internal pseudo-trabeculations and no periosteal reaction (Fig. 1a, b). Magnetic resonance imaging (MRI) showed a large mass with a clear and smooth rim, invading the radial metaphysis and carpal articular surface (Fig. 1c-e). The patient was hospitalized, and tests revealed normal complete blood count, blood coagulation indicators, as well as liver and kidney function. To gather further details about the tumor, the patient underwent a C-arm-guided percutaneous core needle biopsy. A stab incision was made at the radial side of the forearm, and the tumor was sampled using a needle, with angulation and depth monitored via C-arm fluoroscopy. Based on histological examination, fibrous dysplasia was initially suspected; however, low-grade fibrosarcoma was not excluded.

**Fig. 2:**
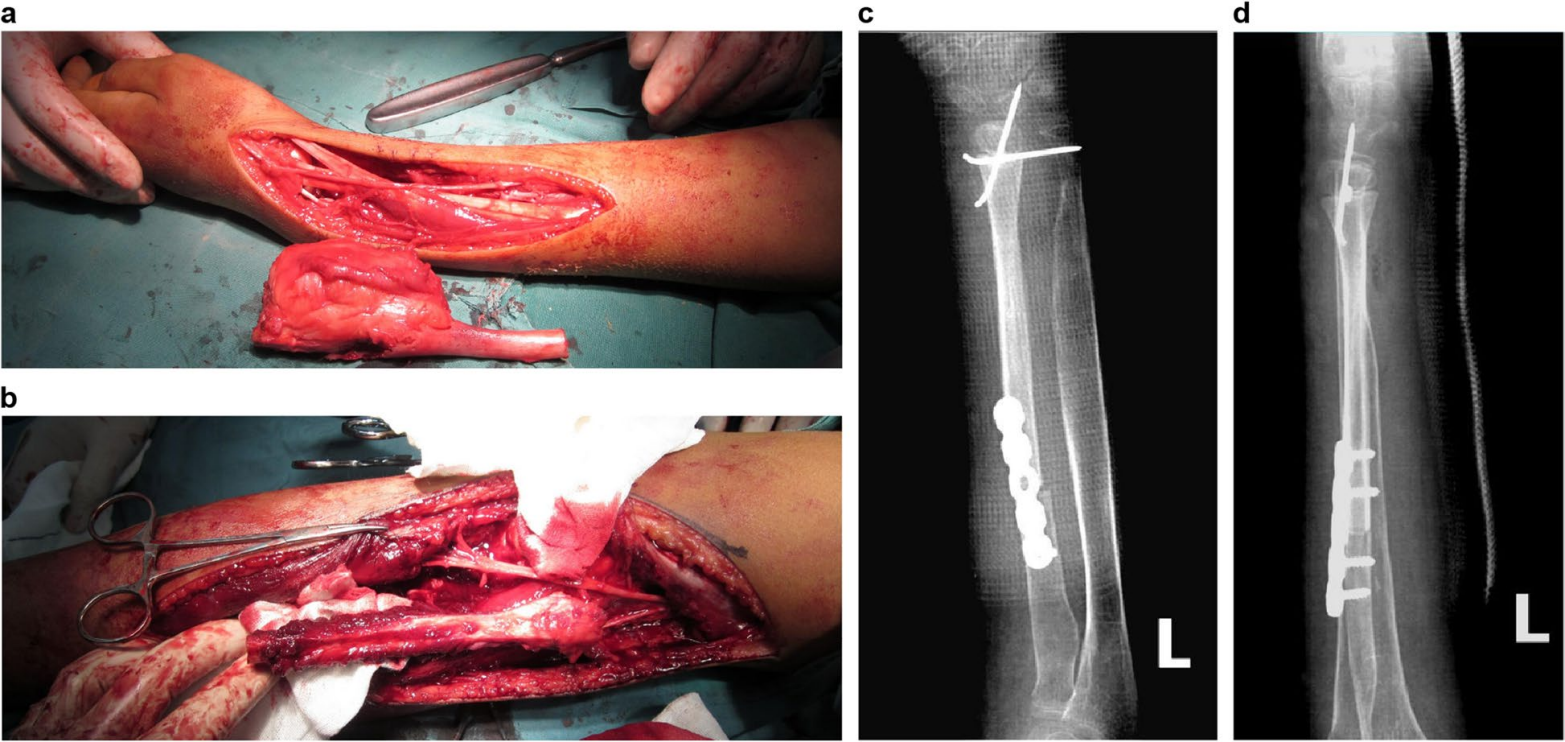
**a** The mass measures approximately 8 × 4 × 4 cm^3^, and the affected radius is excised to approximately 13 cm in length. **b** A 13 cm-long free vascularized fibular proximal epiphyseal graft is harvested from the ipsilateral lower leg. **c**, **d** The fibular graft is fixed with a reconstruction plate and two 2.0 mm Kirschner wires

**Fig. 3:**
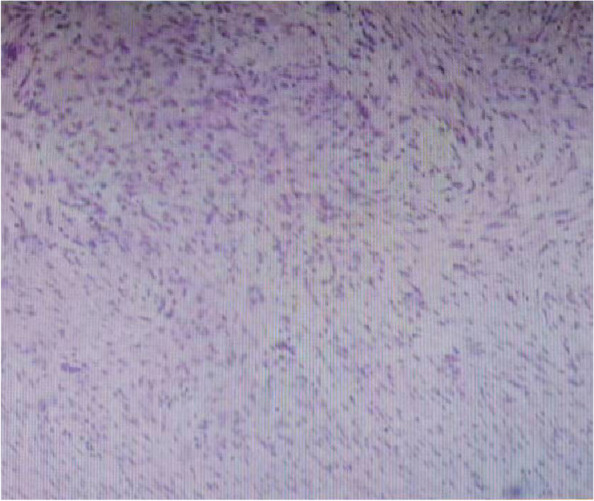
Microscopic examination reveals abundant fusiform fibroblasts and collagen fibers partially infiltrating the surrounding skeletal muscle

To maximize wrist function recovery, we devised a two-stage procedure after extensive discussions with the patient’s parents and obtaining informed consent. Initially, we performed tumor resection via a radial approach. A longitudinal incision was made, facilitating dissection through the soft tissue. Identification and protection of the radial artery, radial vein, and superficial radial nerve were ensured. Subsequently, the lesion and surrounding normal tissues were meticulously exposed. The mass, measuring approximately 8 × 4 × 4 cm^3^, was found to originate from the medullary canal of the distal radius and expanded outward without apparent invasion of the surrounding soft tissues (Fig. 2a). After confirming that the surgical margins were tumor-free, approximately 13 cm of the affected radius was excised (Fig. 2a). We planned to perform a 13 cm-long free vascularized fibular proximal epiphyseal transfer, utilizing the peroneal artery as the vascular pedicle. A lateral incision was made in the ipsilateral lower leg, and dissection was performed carefully to isolate the vascular bundle while protecting the common peroneal nerve. The fibula was separated from the surrounding muscles and osteotomized using a wire saw, leaving the fibula graft attached to the limb solely by the vascular bundle (Fig. 2b). Before severing the peroneal artery, the adequacy of blood supply to the fibular graft solely pedicled on the peroneal artery was confirmed by observing bleeding from both the medullary canal and epiphysis. The peroneal vessel was anastomosed to the radial vessel in an end-to-end fashion. The fibular shaft was fixed to the radial shaft using a reconstruction plate, and the fibular head was fixed to the proximal carpal row and stabilized with two 2.0 mm Kirschner wires (Fig. 2c, d). However, considering the risk of vascular network damage in the epiphysis, ligament reconstruction was not performed. A long splint was applied for additional fixation after surgery, and blood flow in the transferred fibula was monitored using Doppler ultrasound. The complete specimen was sent to the pathology department for comprehensive histological examination, which confirmed a diagnosis of DF (Fig. 3).

**Fig. 4:**
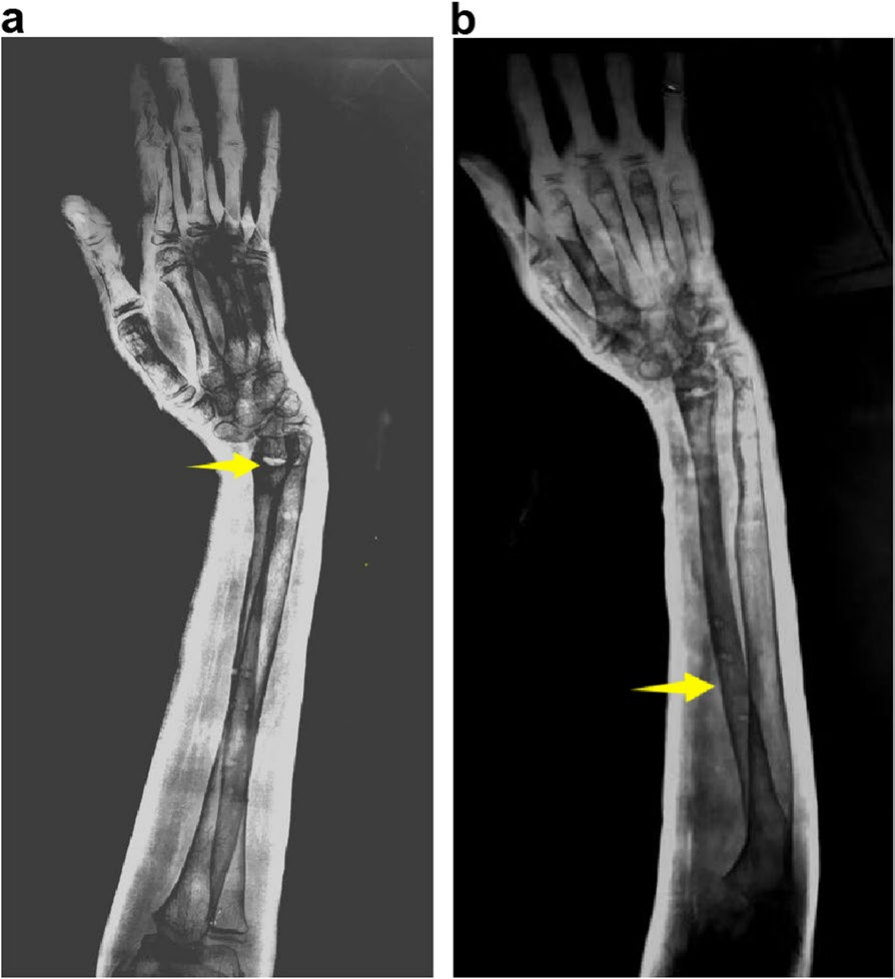
**a** Radiographs showing the growth plate after 12 months (yellow arrow) and **b** bone union (yellow arrow), with radial volar subluxation of the wrist (**a**, **b**)

The bone union between the fibular graft and the proximal radius was achieved after 6 months, and the patient underwent internal fixator removal at another hospital. At the 12-month follow-up, the patient reported mild pronation in his left wrist but no pain. Radiography revealed radial volar subluxation of the wrist (Fig. 4a, b). Due to financial constraints and family circumstances, he did not receive further treatment.

**Fig. 5:**
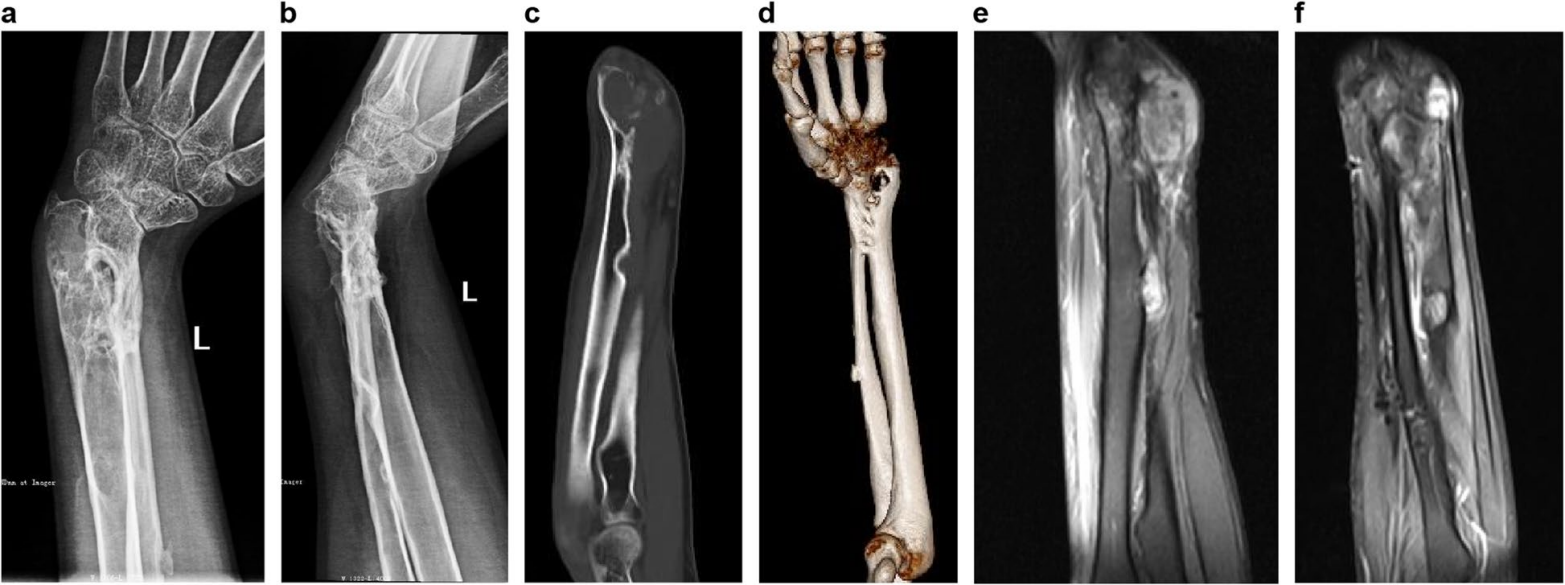
**a** Anteroposterior and **b** lateral radiographs show extensive cystic destruction in the distal ulnar with wrist deformity. **c** Computed tomography (CT) demonstrates expansion and cortical breakthrough of the ulna head accompanied by an extraosseous soft tissue mass. **d** Three-dimensional CT shows synostosis between the fibula graft and ulna. **e**, f Magnetic resonance image reveals an irregular mass with an uneven equal signal invading the distal half of the ulna

**Fig. 6:**
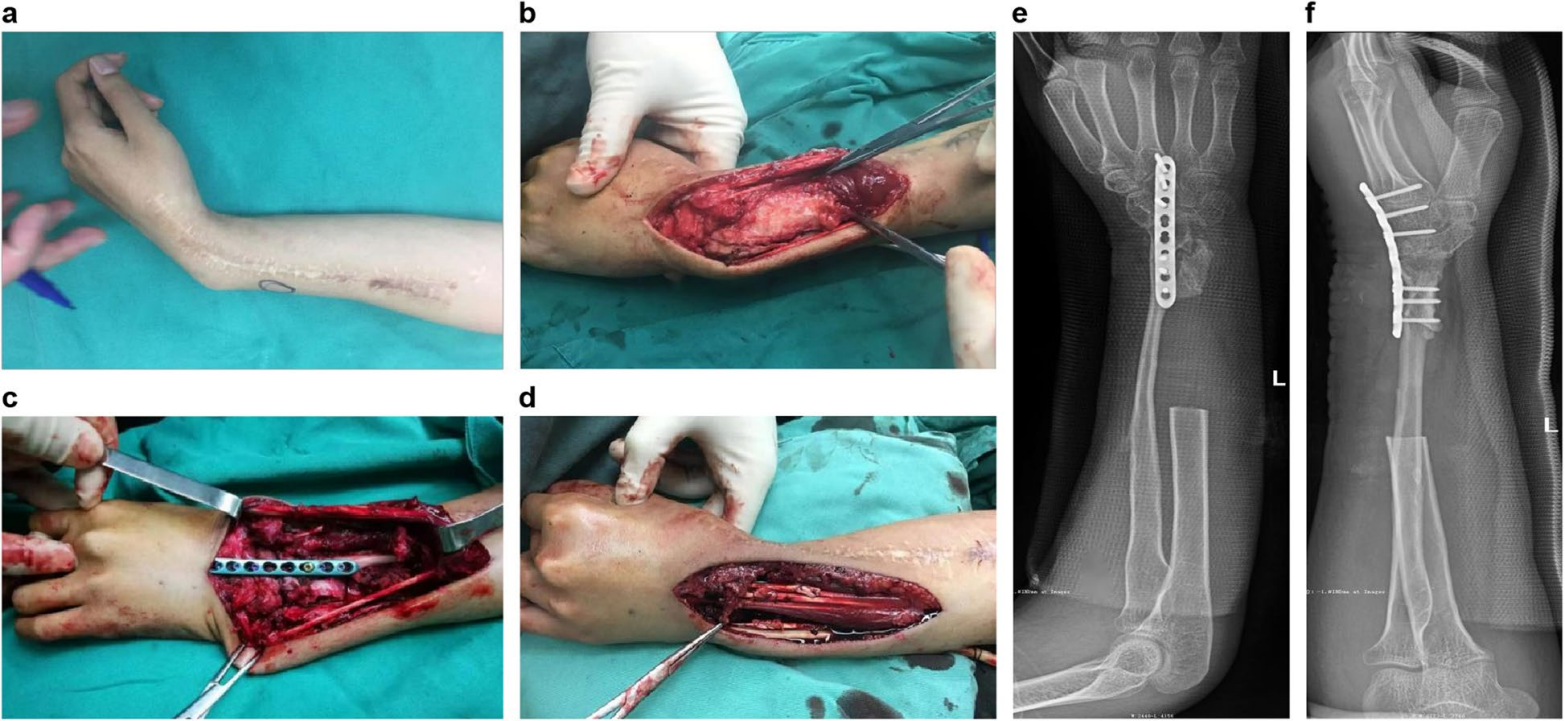
**a** The patient underwent a second tumor resection; **b** an irregular mass with cortical breakthrough is observed; **c** tumor resection and wrist fusion were performed in a single stage; **d** the extensor tendons were cut off and repaired at different levels. **e** Anteroposterior and **f** lateral radiographs showing approximately 10 cm length of the resected affected ulna, the corrected wrist deformity, and a 2.7 mm dynamic compression plate used for wrist fusion

**Fig. 7:**
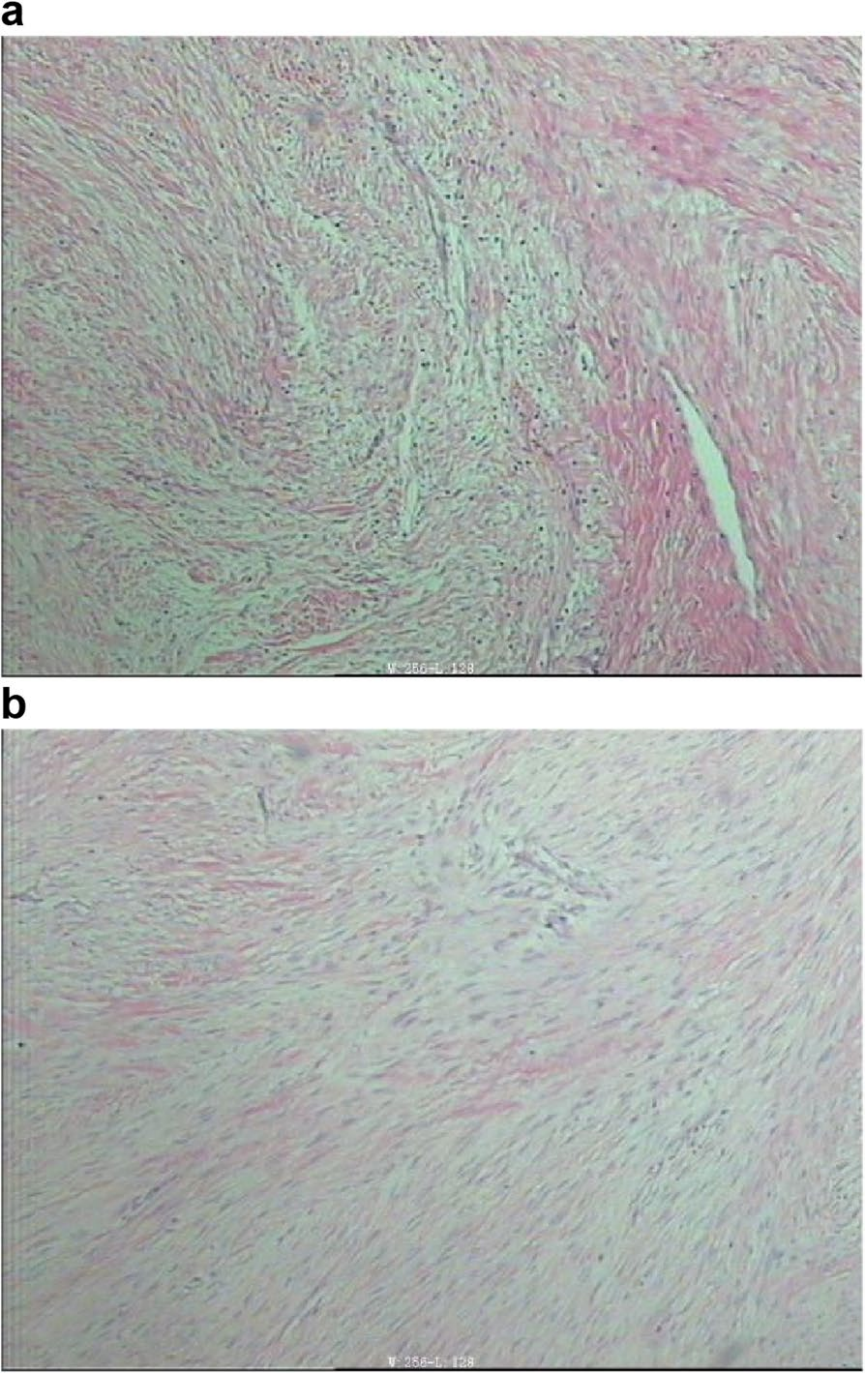
**a**, **b** Histological examination confirmed the recurrence of the desmoplastic fibroma in the ulna, invading the surrounding adipose tissue and skeletal muscle

In February 2019, the patient returned to our hospital because of severe wrist deformity and functional impairment. Physical examination revealed a local mass on the dorsal ulnar side of his left hand, with the wrist fixed in a position of palmar flexion and radial deviation and the range of motion at almost zero. Anteroposterior and lateral radiographs of the left forearm showed extensive cystic destruction of the distal ulna and abnormal wrist joint alignment characterized by radial deviation, palmar subluxation, and joint space narrowing (Fig. 5a, b). Computed tomography (CT) demonstrated expansion and cortical breakthrough of the ulnar head accompanied by an extraosseous soft tissue mass (Fig. 5c). Three-dimensional CT revealed synostosis between the fibular graft and ulna (Fig. 5d). MRI showed an irregular mass with uneven and equal signals invading the distal half of the ulna (Fig. 5e, f). The patient was diagnosed with tumor recurrence and underwent surgery. A dorsal longitudinal incision was made; during the operation, a large irregular soft tissue mass on the ulna was observed, which came from the medullary cavity and eroded the dorsal cortex; the resection range included the mass and approximately 10 cm length of the affected ulna (Fig. 6a, b) and the solid bone between the fibular graft and ulna was preserved to reduce fracture risk. Wrist deformity correction and fibula-scapholunate fusion were performed after tumor resection. The wrist was maintained in the neutral position, and a 2.7 mm dynamic compression plate was used for wrist fusion (Fig. 6c, d, e, f). The extensor tendons became loose after palmar flexion correction and were cut off, tightened, and subsequently repaired at different levels to prevent or decrease adhesion (Fig. 6d). Histological examination supported the diagnosis of DF recurrence in the ulna (Fig. 7a, b).

**Fig. 8:**
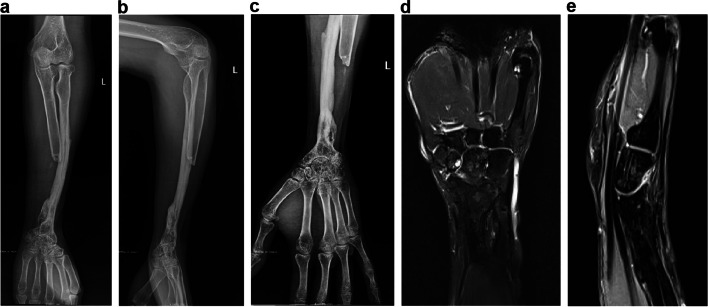
**a**-**c** Radiographs show a stable wrist and elbow and considerable hypertrophy of the fibula graft. **d**, **e** No signs of tumor recurrence are seen on magnetic resonance imaging

**Fig. 9:**
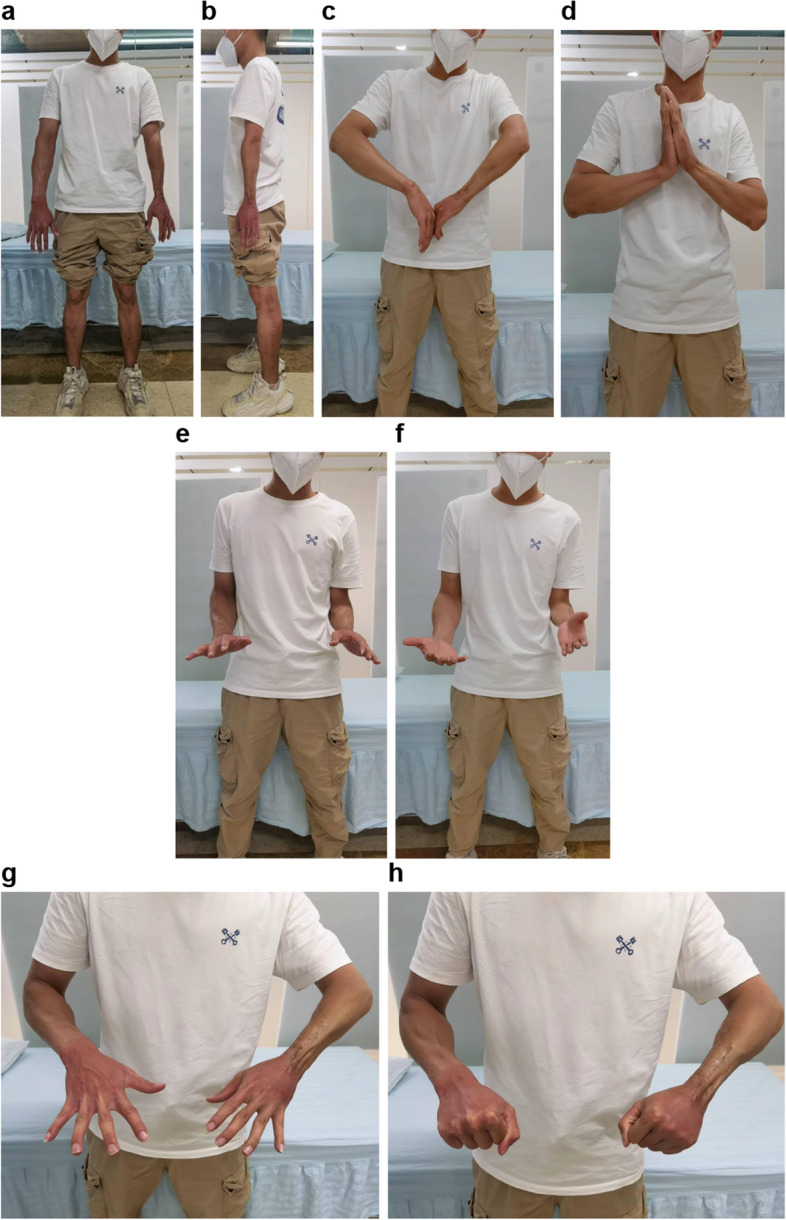
Appearance of the lower limbs in the anteroposterior (**a**) and lateral views (**b**). Range of motion of the (**c**-**f**) wrist and (**g**, **h**) fingers

A splint was applied for protection for at least 6 weeks, and functional finger training was initiated as early as 72 h postoperatively. The surgical incision healed without complications. Radiotherapy, consisting of a total dose of 45 Gy delivered in 25 fractions over 4 weeks, was administered to the left forearm 6 weeks after surgery. Bone union was achieved after 6 months, and the patient underwent internal fixator removal to restore midcarpal joint movement. At the 2023 follow-up after the second tumor resection, radiographs showed a stable wrist and considerable hypertrophy of the fibula graft, which had been remodeled and resembled the radius (Fig. 8a-c). MRI revealed no signs of tumor recurrence (Fig. 8d, e). The patient experienced no pain in the wrist or elbow, and upper extremity function was deemed acceptable (Fig. 9a-h). He expressed satisfaction with the surgical outcomes and said he could work in a factory setting.

## Discussion and conclusions

DF typically lacks specific clinical symptoms, necessitating pathological confirmation for diagnosis. Ensuring accurate diagnosis before surgery is crucial in managing this condition effectively.

Open biopsy is considered the gold standard method; however, there exists a risk of sampling error if the specimen obtained is insufficient or fails to capture the representative area of the lesion. In this case, the patient underwent a core needle biopsy, leading to an incorrect diagnosis of fibrous dysplasia. We speculated that the specimen was obtained from the internal pseudo-trabeculations of the tumor, which resulted in the misdiagnosis. An accurate diagnosis was obtained after the complete specimen underwent pathological examination.

A DF is considered the bony counterpart of the more common desmoid-type fibromatosis. The histologic features of DF are essentially indistinguishable, particularly when the tumors involve both bone and soft tissues [10]. Hauben et al. performed DNA sequencing in 13 DF cases and revealed that activating b-catenin gene mutations were absent, suggesting that the b-catenin pathway may not play a crucial role in DF tumorigenesis, unlike in desmoid-type fibromatosis [11]. However, additional studies are required to confirm the effectiveness of this method. Clinical medical history and imaging findings are key factors for differentiation, and they are mainly based on the tumor’s development, size, and invasive form. Desmoid-type fibromatosis occurs in soft tissues, often resulting in compressive invasion and superficial cortical defects. By contrast, DF arises in the bone and presents with osteolytic lesions and destruction of the cortex accompanied by relatively clear boundaries of the soft tissue mass. Radiography, CT, and MRI should be performed for tumor assessment. Zhang et al. suggested that DF should be considered when multilocular or “whisker-like” tumor bone trabeculae are observed on plain radiography and CT, with no significant enhancement on contrast-enhanced scans and a patchy low signal on both T1-weighted images and T2-weighted images of MRI sequences [3]. Additionally, the absence of periosteal reaction, calcification, or ossification suggests DF [3].

DF must be differentiated from other malignant tumors, particularly fibrosarcoma and low-grade intraosseous osteosarcoma, as well as low-grade fibrosarcoma. The distinction between DF and low-grade fibrosarcoma can be particularly challenging. In most cases, fibrosarcomas exhibit high cell density, high-grade polymorphisms, and high mitotic rates. However, differentiating between the two tumors can be difficult when a low-grade fibrosarcoma presents with predominant collagen tissues, or DF exhibits dense cellularity and soft tissue invasion. In such cases, long-term follow-up is required to confirm the final diagnosis [4]. The malignant tendency of DF remains unclear. A limited number of case reports have suggested the possible transformation of DF into osteosarcoma. However, these findings should be interpreted cautiously as the initial diagnosis of DF in these cases might have been erroneous.

The treatment of DF remains controversial at present, and most surgeons recommend surgical treatment according to the tumor’s location and extent. Treatment options include wide resection, intralesional resection, or curettage. Amputation should be avoided unless malignant degeneration or metastasis is present. Evans et al. reported that of the six patients who underwent intralesional curettage, two patients with extraosseous soft tissue components developed rapid local recurrence, suggesting that extraosseous tumor growth is a negative prognostic factor and requires more radical surgery [5]. Böhm et al. analyzed 191 published cases of DF and found that the recurrence rate was 55–72% following non-resection procedures compared with a significantly lower recurrence rate of 17% after resection. DF cases located in the extremities had a recurrence rate of 55% (32/58), and 25% (8/32) of these cases required amputation [9].

To reduce recurrence, the patient underwent wide resection surgery, which was not technically challenging. However, wrist reconstruction was challenging. Various procedures have been used for wrist reconstruction, including prosthetic replacement, arthrodesis, single-bone forearm formation, nonvascularized or vascularized fibular grafting, and allograft replacement. Prosthetic replacement is not suitable for small children and cannot fulfill the requirements of long-term viable reconstruction for survival over decades [12]. Avascular autografts or allografts have a high risk of nonunion, delayed union, and even fractures and apply to defects < 6 cm in size [13]. Arthrodesis or single-bone forearm formation can result in a stable, pain-free wrist; however, it may lead to loss of wrist mobility [14]. Vascularized fibular proximal epiphyseal graft contains an articular surface similar to the distal part of the radius and has the potential for growth and remodeling over time; therefore, it has been considered an ideal procedure for wrist reconstruction in children. Innocenti et al. reported about six patients with a mean age of 8.4 years who presented with malignant bone tumors in the distal part of the radius. Following tumor resection, they underwent microsurgical reconstruction of the distal part of the radius using vascularized proximal fibular transfers, with graft lengths ranging from 7 to 13.5 cm. The grafts were supplied by the anterior tibial vascular network. Five of six patients had consistent and predictable longitudinal growth. The remodeling of the articular surface of the fibular epiphysis was satisfactory, and the functional outcomes were excellent [15]. Onoda et al. suggested that in cases where the bone defect extends farther than the proximal two-thirds of the diaphysis of the fibula, a bipedicled reconstruction should be considered. Additionally, when the bone defect extends beyond the reach of the tibial vascular supply, the peroneal vessels can be utilized for vascularization [16].

We selected the peroneal artery as the feeding artery due to the large bone defect and the patient’s older age. The anterior tibial vasculature might have provided insufficient blood supply to the fibular shaft, increasing the risk of bone union between the transferred and residual bone. Furthermore, bleeding from the fibular head and medullary canal was confirmed after microvascular anastomosis. At the 12-month follow-up after surgery, bone union was obtained between the fibular graft and the radius, and neither a degenerative change of the radiocarpal joint nor premature fusion of the growth plate was observed, which confirmed sufficient blood supply from the anastomosed vessels.

Vascularized fibular proximal epiphyseal transfer offers a promising approach for pediatric extremity reconstruction, enabling single-stage skeletal and joint reconstruction while minimizing limb discrepancies. However, this technique is technically demanding and carries a risk of complications. Kurlander et al. reviewed 53 patients who underwent upper extremity reconstruction using vascularized fibular proximal epiphyseal transfer [17]. The most common complication encountered was fracture. Impaired function was observed in 75% of patients, partial growth in 31%, and no growth in 4%. Secondary surgery was required in 22% of patients, with flap salvage being the most frequent secondary procedure (7%) [17]. In the present case, the complications, including dysfunction and wrist deformity, were caused by tumor recurrence in the ipsilateral ulna and incongruity between the fibular head and proximal carpal row. In this situation, complex microsurgical reconstruction surgery was no longer the first choice after tumor resection. We performed a wide resection of the affected ulna to remove the tumor and improve the rotational function of the arm. Good stability and appearance were obtained by partial carpal fusion after wrist deformity correction, and a single-stage simple procedure effectively addressed multiple complications and tumor recurrence.

The role of radiotherapy remains uncertain; some reports have shown that radiotherapy is an effective treatment in situations where surgery is not feasible or when a DF invades the soft tissue and wide resection cannot be performed [18, 19]. A recent report revealed that combined surgery and brachytherapy could represent a novel radical approach with reduced trauma and risk [20]. However, some surgeons are concerned about the detrimental effects of radiation on growth and the possible increased risk of malignancy [21]. Given the tumor’s invasion of surrounding soft tissues, the patient underwent adjuvant radiotherapy after surgery. The patient obtained acceptable function and no evidence of tumor recurrence was noted in over 4 years of follow-up.

In conclusion, DF is a rare primary bone tumor, and it is necessary to comprehensively evaluate its clinical, imaging, and histological features to obtain a correct diagnosis. Surgical resection is the main choice of treatment, with radiotherapy being an effective supplementary treatment; however, complex reconstructive surgery and high recurrence rates make treatment challenging. The reconstruction of large skeletal defects in children after tumor resection is a unique challenge, as the free vascularized fibular proximal epiphyseal transfer is a more reliable method for biological reconstruction of the wrist; however, it has high technical requirements and common complications, and experience in reconstructive surgery and careful follow-up is required for excellent long-term functional results.

## Data Availability

All the data are available from the corresponding author upon reasonable request. Written informed consent is obtained from the participants and/or their parents/legal guardians for publication of the case report.

## Abbreviations

DF: Desmoplastic fibroma
MRI: Magnetic resonance imaging
CT: Computed tomography
